# Twitter Usage Among Physicians From 2016 to 2020: Algorithm Development and Longitudinal Analysis Study

**DOI:** 10.2196/37752

**Published:** 2022-09-06

**Authors:** Keisuke Nakagawa, Nuen Tsang Yang, Machelle Wilson, Peter Yellowlees

**Affiliations:** 1 Department of Psychiatry and Behavioral Sciences University of California, Davis School of Medicine Sacramento, CA United States; 2 Digital CoLab Innovation Technology University of California, Davis Health Sacramento, CA United States; 3 Division of Biostatistics Department of Public Health Sciences University of California, Davis Sacramento, CA United States

**Keywords:** social media, internet, health informatics, internet use, public health, Twitter, physician

## Abstract

**Background:**

Physicians are increasingly using Twitter as a channel for communicating with colleagues and the public. Identifying physicians on Twitter is difficult due to the varied and imprecise ways that people self-identify themselves on the social media platform. This is the first study to describe a reliable, repeatable methodology for identifying physicians on Twitter. By using this approach, we characterized the longitudinal activity of US physicians on Twitter.

**Objective:**

We aimed to develop a reliable and repeatable methodology for identifying US physicians on Twitter and to characterize their activity on Twitter over 5 years by activity, tweeted topic, and account type.

**Methods:**

In this study, 5 years of Twitter data (2016-2020) were mined for physician accounts. US physicians on Twitter were identified by using a custom-built algorithm to screen for physician identifiers in the Twitter handles, user profiles, and tweeted content. The number of tweets by physician accounts from the 5-year period were counted and analyzed. The top 100 hashtags were identified, categorized into topics, and analyzed.

**Results:**

Approximately 1 trillion tweets were mined to identify 6,399,146 (<0.001%) tweets originating from 39,084 US physician accounts. Over the 5-year period, the number of US physicians tweeting more than doubled (ie, increased by 112%). Across all 5 years, the most popular themes were general health, medical education, and mental health, and in specific years, the number of tweets related to elections (2016 and 2020), Black Lives Matter (2020), and COVID-19 (2020) increased.

**Conclusions:**

Twitter has become an increasingly popular social media platform for US physicians over the past 5 years, and their use of Twitter has evolved to cover a broad range of topics, including science, politics, social activism, and COVID-19. We have developed an accurate, repeatable methodology for identifying US physicians on Twitter and have characterized their activity.

## Introduction

Twitter has become a popular communication tool, with almost 200 million daily active users and more than 500 million tweets being sent out every day [1]. Around the world, people have used Twitter as a platform for sharing information, expressing opinions, and engaging in social movements. It has democratized communication, allowing everyday citizens to have a voice at the same potential scale as the voices of global leaders, politicians, and celebrities. The speed at which information is shared on Twitter has transformed the way information spreads and how communities create social movements. Studies have shown that Twitter is one of the best sources of real-time information, outpacing traditional media outlets for reporting natural disasters, crimes, and major events [2-6].

Over the past 15 years, physicians have started to use Twitter as a platform for sharing scientific information, frontline experiences, and opinions on various topics [7-12]. Studies have shown that scientific papers obtain more visibility when shared on Twitter, and impact metrics have started to incorporate social media data as part of their calculations [13]. During the COVID-19 pandemic, Twitter has given unprecedented visibility to clinicians working on the front lines. Clinicians have engaged in public discourse by sharing data and scientific information on Twitter [14-17]. They have also engaged in dialogue with each other, often sharing encouragement, empathy, and compassion during challenging times [18,19].

Studies have already demonstrated the use of Twitter in building early warning systems for adverse drug reactions, understanding discussions about various diseases, and characterizing public perceptions regarding COVID-19 [20-24]. One of the earliest uses of Twitter among physicians was tweeting during conferences, and a number of studies have analyzed what factors make tweets more successful or more likely to be disseminated during medical conferences [9,25]. Recent studies have demonstrated that physician activity on Twitter is a potential predictor of COVID-19 surges [26]. As more physicians join Twitter and engage in dialogue across a wide range of topics, health systems and academic medical centers that train and often employ these physicians need to be aware of these trends to teach them about professionally appropriate use and potential repercussions. Physicians should be aware of the positive and negative consequences of engaging in public discourse on platforms such as Twitter, where content is instantly made public and is often permanent and irreversible [23,27].

Although some studies have investigated the use of Twitter among physicians in narrow use cases, such as a specific specialty, no study has characterized the overall activity of US physicians on Twitter across multiple years [11]. Part of this can be attributed to the difficulty of identifying physicians on Twitter. Physicians self-report their professional status in varied, inconsistent, and sometimes vague ways, and there are limited methods for verifying and validating the identity of a user based on their profile and tweet content. Furthermore, the challenges of combing through billions of tweets and bios to identify physician accounts can be computationally and logistically intensive. It is for these reasons that a process for identifying US physicians and characterizing their activity has not been published, to our knowledge. Given the widespread use of Twitter by the general public and among physicians, it is becoming increasingly important to understand the use of social media among physicians, particularly with the backdrop of the COVID-19 pandemic. The aims of this study are to describe a reliable and repeatable methodology for identifying US physicians on Twitter and to characterize their use of Twitter and longitudinal trends across multiple years.

## Methods

### Process for Identifying US Physicians on Twitter

**Figure 1:**
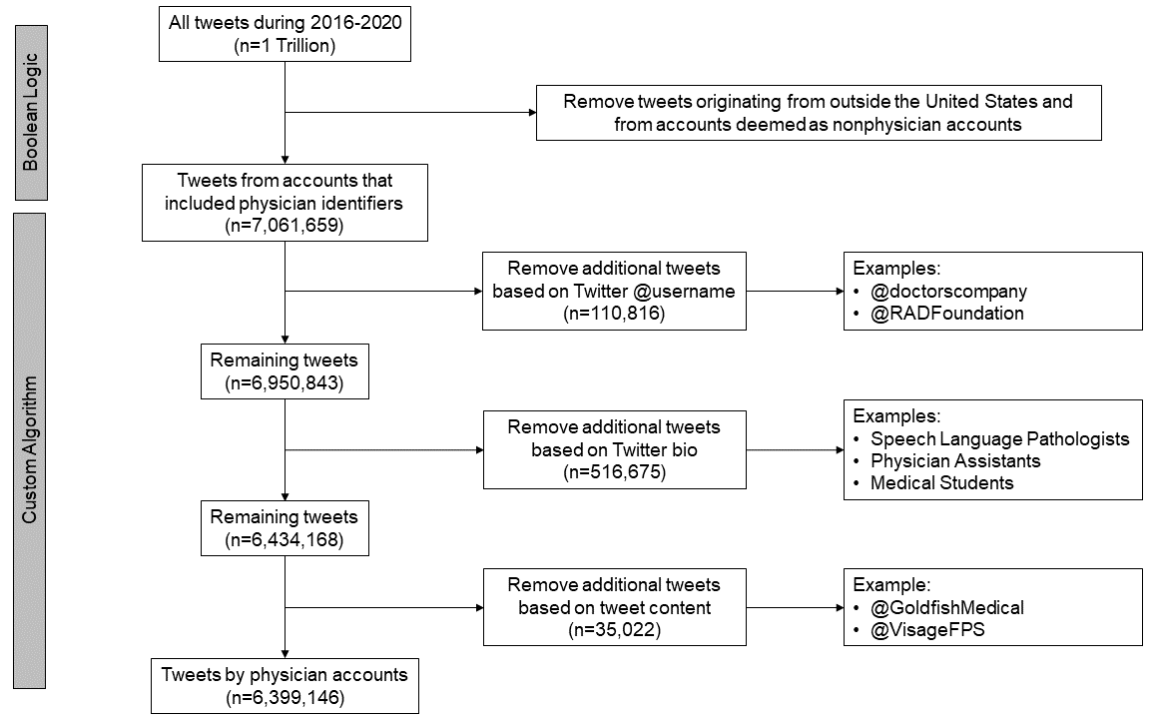
Identification and selection of physician tweets.

We conducted an in silico analysis of physicians’ Twitter usage over a 5-year period. We identified physicians by using a 3-stage process (Figure 1) comprising a geographic filter for excluding accounts from outside of the United States, Boolean logic for further identifying US physician accounts based on their usernames and bios, and a custom algorithm for filtering out false-positive accounts. The details of the algorithm developed and the iterative refinement process are described in the following sections.

### Algorithm Development and Refinement for Physician Identification

Initially, a Boolean logic was developed (Multimedia Appendix 1) to scan all Twitter usernames (ie, *@username*), names (eg, *Jane Doe, M.D.*), and bios for every tweet that was posted during the 5-year study period (2016-2020). The Boolean logic identified all tweets that included some indication of a Twitter user’s status as a physician (eg, mentioning *physician* in the bio or including a medical degree, such as *M.D.* or *D.O.*, in the name or username). The Boolean logic included a corpus that contained every specialty and subspecialty listed by the Accreditation Council for Graduate Medical Education and the American Osteopathic Association. A third-party software (Meltwater Explore; Meltwater, Inc) was used to review the user accounts and tweets generated from the Boolean algorithm to ensure the accuracy of the Boolean logic expression. With each iteration, the first 1000 tweets were manually reviewed for any tweets that did not originate from a physician-owned account. Appropriate logic was added to filter out false positives. Examples of false positives are provided in Table 1. This manual review process was repeated until the false-positive rate decreased to less than 5% in a 1000-tweet sample.

**Table 1:** Examples of false positives and the associated Boolean logic.

Description	Examples	Boolean logic
*MD* is used in the username, in the profile, or in both to indicate something other than a medical degree.	*MD* in “@MDZeki” stands for “Muhammad” *MD* in “Student living in MD” and “@MDJobs” stands for “Maryland”	Only identify tweets that are in English and from the United States Only include usernames that have “,M.D.,” “,MD,” or “, MD” Remove all accounts that include *jobs* in the username or profile
*DO* is used in the username, in the profile, or in both to indicate something other than a medical degree.	*DO* in “@JUSTDOIT” does not indicate a DO medical degree	Only include names that have “,D.O.”
*Doctor* is used broadly without specifying an MD or DO degree.	@DoctorJones @aprokodoctor @DoctorsEMres	The Boolean logic did not select handles with *Doctor*; the user would have had to indicate their status as a physician through more specific ways
Premedical and medical students who include *physician* or *doctor* in their username or profile.	Profile includes “Aspiring Infectious Disease Physician” Profile includes “aspiring physician working in epi & vaccine research” Profile includes “future physician” or “Future Physician Assistant”	Remove all accounts that include *aspiring* or *future* in the profile

### Data Acquisition

Once the Boolean logic was finalized, all tweets that were written in English; originated from the United States; were posted between January 1, 2016, to December 31, 2020; and met the Boolean criteria were acquired from a third-party vendor (Meltwater, Inc, San Francisco, California).

### Algorithm Refinement

The data were imported into RStudio (Version 1.2.5033; RStudio, PBC), where additional rules were programmed in R (R Foundation for Statistical Computing) and applied to filter out nonphysician Twitter accounts. The next phase of algorithm refinement involved filtering out more nuanced cases that the Boolean logic could not catch. Accounts that used *MD* to indicate the state of Maryland; accounts of physician recruiting services that used variations of *MD* and *jobs* in their usernames and profiles; and premedical and medical students who used *aspiring MD*, *aspiring physician*, *future physician*, or similar terms in their profile were filtered out by using logic coded in R.

The algorithm was further refined by identifying patterns in the tweet text itself and then applying filtering logic to the accounts of those tweets. For example, to filter out more physician recruitment accounts, tweets that used the hashtags *#job* or *#jobs* in the text were identified. Among these tweets, any accounts that included the word *medical* or *surgery* in the account name were eliminated (eg, *Goldfish Medical* or *Visage Facial Plastic Surgery*). This 2-step process of screening accounts—analyzing the tweet text first and then identifying false-positive usernames and names—was used to filter out the final batch of nonphysician accounts.

### Algorithm Accuracy and Validation

We used Twitter’s verified accounts (ie, those with a blue badge) as the validation set to test the accuracy of the algorithm. Twitter uses a rigorous verification process to confirm the identities of verified account users, which includes checking for an official government-issued identification document or an official email address [28].

All of the tweets that originated from a verified Twitter account were separated from the larger 5-year data set. A team manually reviewed each unverified account by visiting the associated Twitter profiles and searching the internet to confirm the details of a user’s status as a physician (eg, checking the website of their stated institution for their profile). A false-positive error rate was calculated based on the number of verified accounts that belonged to Twitter users who were not actually physicians. In order to calculate the false-positive rate of unverified accounts, around 5% (1978/43,328, 4.6%) of the unverified accounts in the data set were randomly selected. The accounts were manually reviewed by the team to calculate a false-positive rate for the unverified physician accounts identified by the algorithm.

### Hashtag and Topic Analysis

The top 100 hashtags used by US physicians were identified, tallied, and ranked for each year. Each tweet was tokenized, stemmed, and normalized, and stop words were removed. This was done by using the following R packages: *stringi*, *stringr*, and *tokenizers*. All words that followed a pound sign were identified and separately stored within a data frame in RStudio. For every tweet, each hashtag was counted and tallied across all tweets for each year (2016-2020). Researchers (KN and NTY) reviewed the list and grouped any terms that were related. For example, the COVID-19 topic was comprised of hashtags such as *#covid19*, *#coronavirus*, *#sarscov2*, *#covid19coronavirus*, and other related hashtags. Further, both the *#blacklivesmatter* and *#blm* hashtags were used for the Black Lives Matter topic’s tally and rank.

We analyzed hashtags in two ways. First, we created word clouds for a visual representation of Twitter activity. A word cloud was generated for each year of the study period; words were color-coded based on their frequency of use. Word clouds were generated by using the following R packages: *wordcloud*, *wordcloud2*, and *ggplot2*. Second, we identified the top 100 hashtags and categorized them into broad thematic areas. In order to analyze thematic trends between years, hashtags were organized into the following themes: general health care, COVID-19 and public health, politics, social activism, mental health and well-being, health technology, conferences, patient groups, specialties and subspecialties, medical conditions and procedures, and medical education. For the general health care theme, tweets that included generic health care–oriented hashtags like *#healthcare*, *#doctor*, *#medtwitter*, and *#medicine* were combined and tallied, and the proportion of such tweets was calculated as a percentage of all tweets for each year. For the COVID-19 and public health theme, hashtags like *#covid19*, as well as hashtags that conveyed public health messages like *#maskup*, *#socialdistancing*, and *#flattenthecurve*, were included. For the politics theme, any hashtag related to politics and elections, such as *#trump*, *#vote*, *#election2020*, *#tcot* (ie, top conservatives on Twitter), and *#newsmax*, were used [29,30]. Only the top 100 hashtags for each year were considered.

### Ethical Considerations

No application for an ethics review board assessment was submitted, since this study involved third-party data sets with no experimental activities. Therefore, this study was deemed a quality assurance/quality improvement activity [31].

## Results

### Algorithm Accuracy

Of the 216 Twitter-verified accounts that the Boolean algorithm identified as physician accounts, 12 were not physician accounts, resulting in an accuracy rate of 94.4% (204/216). Of the 43,328 unverified accounts in the data set (ie, regular Twitter accounts), 1978 accounts were randomly selected for manual review as part of the 5% validation sample. Of the 1978 randomly-selected unverified accounts, 204 were not physician accounts, resulting in an accuracy rate of 89.7% (1774/1978; 95% CI 88.3%-91%).

### Twitter Activity

The number of US physicians who were active on Twitter increased from 2016 to 2020. The number of unique physician accounts tweeting increased from 12,675 in 2016 to 26,897 in 2020—a 112.2% increase in the number of active physician users over the 5-year period (Table 2). The number of new physician accounts per year also increased from 1711 new accounts in 2016 to 2215 new accounts in 2020—a 19.7% year-over-year increase in the number of new physician accounts created. Although the total number of US physician accounts increased, the total number of tweets varied across the 5-year period, with 1,461,753 total physician tweets in 2016 and 1,338,150 total physician tweets in 2020. The average number of tweets per account decreased from 115.3 tweets per physician account in 2016 to 49.8 tweets per physician account in 2020. These trends were also reflected in a subgroup analysis of verified Twitter accounts.

**Table 2:** Twitter activity among US physicians from 2016 to 2020.

Twitter activity		2016	2017	2018	2019	2020
**US physicians on Twitter**						
	Tweets per year, n	1,461,753	1,344,911	1,205,053	1,049,279	1,338,150
	Unique accounts tweeting, n	12,675	15,633	17,934	20,584	26,897
	New accounts tweeting, n	1711	2170	1917	1760	2215
	Number of tweets per account, mean	115.3	86.0	67.2	51.0	49.8
**Verified Twitter accounts^a^**						
	Tweets per year, n	57,255	56,082	49,743	51,776	77,609
	Unique accounts tweeting, n	107	132	155	171	208
	New accounts tweeting, n	2	6	3	8	2
	Number of tweets per account, mean	535.1	424.9	320.9	302.8	373.1

^a^Verified accounts have a blue verified badge next to the Twitter user’s name to let people know that an account is authentic and has undergone rigorous verification by Twitter. More information is available on the Twitter Help Center [28].

### Hashtag and Topic Analysis

Among US physicians using hashtags in their tweets, *#health*, *#healthcare*, *#doctor*, *#medicine*, *#meded*, *#mentalhealth*, and *#wellness* consistently ranked among the most frequently used hashtags across all 5 years of the study period (Multimedia Appendix 2). In 2016, *#digitalhealth* and *#realdonaldtrump* were trending hashtags, while *#trump*, *#backfiretrump*, *#diversity*, and *#inclusion* were trending in 2018 and 2019. Hashtags related to *#blacklivesmatter* and *#covid19* represented 0.3% (3750/1,291,567) and 10.6% (137,107/1,291,567) of all hashtags used in 2020, respectively. Donald Trump–related hashtags were consistently ranked among the top 50 hashtags from 2016 to 2019, and in 2020, US physicians used COVID-19–related hashtags in 137,107 tweets, making them the most used hashtags during the entire 5-year study period (Multimedia Appendix 2), with *#health* being the second most used hashtag across all 5 years (used in 64,092 tweets).

The word clouds provide a visual representation of the frequency and distribution of the hashtags used each year. The frequency of words was color-coded; gray represents the most frequently used hashtags, green represents the least used hashtags, and other colors (light green, pink, purple, and orange) represent the different levels of frequency in between the two extremes. Multimedia Appendix 3 shows the word clouds for tweets from 2016 to 2019. The word clouds for 2016 to 2019 show a broad distribution of words, as demonstrated by the wide variety of colors and the varying sizes of words in the word clouds (Multimedia Appendix 3). The 2020 word cloud shifts drastically, showing just 1 hashtag in gray (*#COVID-19*) and 1 hashtag in orange (*#medtwitter*); all other hashtags are in green (ie, the least used hashtags).

**Figure 2:**
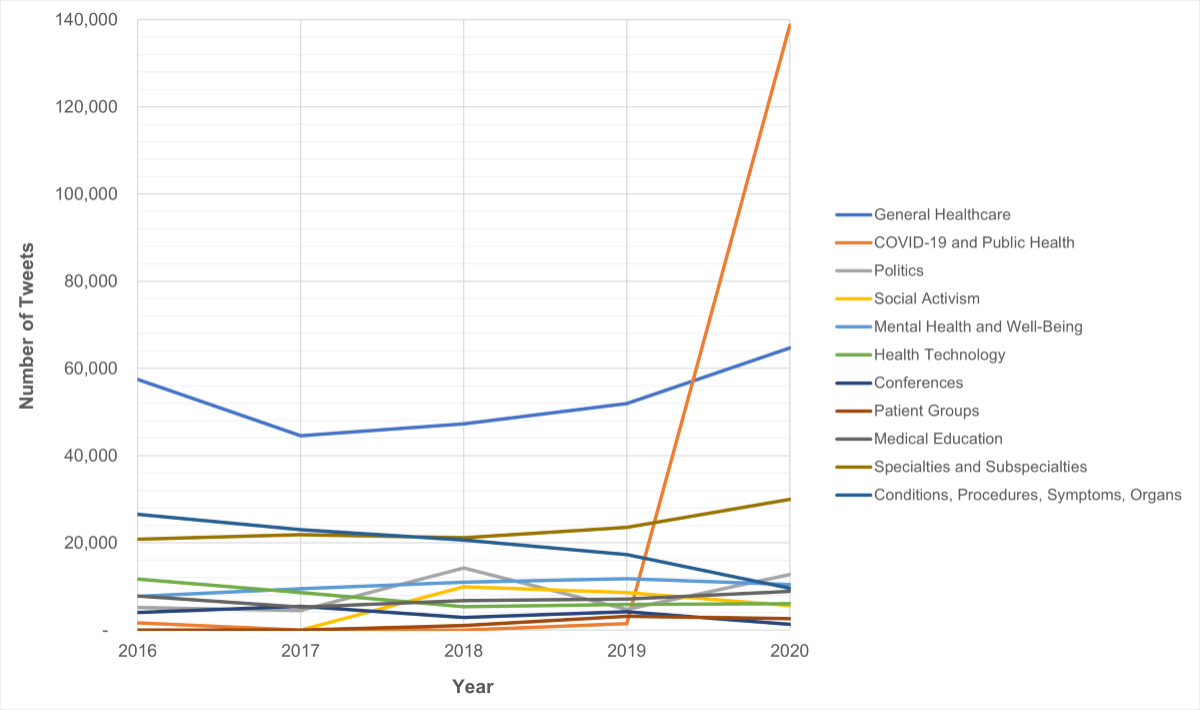
Trending themes by year.

Once hashtags were organized into themes, the results showed that the general health care topic was the most consistently discussed topic across all 5 years and represented 5% (64,701/1,291,567;2020) of the total tweets in 2020 (Figure 2). In approximately 2% (29,964/1,291,567;2020) of tweets, US physicians discussed specialties and subspecialties, and 0.5% (8830; 1,291,567;2020) of tweets discussed medical education. Public health topics were discussed in less than 0.5% (1478/1,186,835; 2019) of the tweets from 2016 to 2019, but in 2020, US physicians discussed COVID-19 and related public health messages—the most dominant theme across all 5 years of the study period—in over 10% of all tweets.

## Discussion

### Study Overview

This is the first longitudinal report of physician activity on Twitter. Physician activity on Twitter has been increasing over the past 5 years, with more physicians creating accounts and joining the platform every year. The diverse range of topics that are discussed on the platform, from social activism and politics to memes, demonstrate that Twitter is becoming a mainstream communication tool for everyday physicians.

The trends in the last 5 years likely indicate that the early adopters phase of Twitter use among physicians is sunsetting, and Twitter is becoming a mainstream platform for physicians [32]. This is supported by the decreasing average number of tweets per user, which shows that the fraction of power users, that is, users with a typically high number of tweets per year, is decreasing in the population of US physician users while use by mainstream physicians, who tend to have a lower number of tweets per year, is increasing. The best example of this occurred in 2020, which was when the content topics drastically shifted toward the topic of COVID-19—a mainstream issue of interest among physicians and the public. Similar trends were seen during the election years (2016 and 2020) and around the time of the Black Lives Matter movement in 2020. If Twitter use among physicians was still in the early adopters phase, we would expect to see less volatile shifts in topics from year to year, since the interests of a niche group would be more stable and consistent.

We also want to acknowledge the role of misinformation and disinformation on the internet. Although this was not the focus of this study, this topic is inextricably linked to the roles and responsibilities that physicians have on Twitter, social media, and the internet. The study period included the 2016 US presidential election and the COVID-19 pandemic, of which both involved the sharing of misinformation and disinformation related to these events [33-36]. Physicians who are active on social media have a disproportionately large influence on the public discourse around science, social movements, and politics, which is supported by the findings in this study. We anticipate that this trend will only continue to increase, placing more importance on studying and understanding the role that physicians play on social media in shaping society’s perceptions and opinions.

### Principal Findings

Physicians discuss a broad range of topics on Twitter and are not shy about opining on topics that are relevant to society beyond strictly medical topics. Physicians are unlikely to find an equivalent platform (traditional or social media) where ordinary clinicians’ voices can be shared and heard globally in such a public, transparent way. This was illustrated by the outsized influence that physicians like Bob Wachter (@Bob_Wachter), physician influencers like Kevin Pho (@kevinmd), and ordinary clinicians have had by using Twitter during the COVID-19 pandemic. Among the hundreds of millions of tweets shared in 2020 [1], the 137,107 (<0.001%) physicians tweeting about COVID-19 may have had a disproportionately outsized impact on public dialogues and disease perceptions.

US physicians are increasingly using Twitter to teach and share medical education materials. Hashtags like *#meded* and *#foamed* (ie, free open-access medicine) were consistently ranked in the top 10 and top 100 hashtags, respectively (Multimedia Appendix 2). This demonstrates a growing shift toward the democratization of medical education through the sharing of materials, study sheets, and illustrations. This shift toward democratizing medical education is likely to continue, with Twitter and other social media platforms, such as Instagram, playing an increasing role in the future of medical education.

Tweeting during medical conferences continues to be a mainstay of use cases among US physicians, but it has become less prominent relative to more mainstream topics. The American Academy of Pediatrics, American Society of Clinical Oncology, and American College of Cardiology conference hashtags (eg, *#aap18*, *#acc19*, and *#asco20*, respectively) were ranked in the top 100 hashtags across the majority of the 5-year study period (Multimedia Appendix 2). Social media continues to be a powerful tool for disseminating scientific information through medical conferences and scientific journals.

Another major trend in Twitter activity was its growing use for marketing purposes. Hashtags like *#healthcaremarketing*, *#medicalmarketing*, and *#plasticsurgery* were consistently ranked in the top 50 hashtags (Multimedia Appendix 2). During the filtering process, a large group of accounts had to be removed that were associated with physician recruitment services using hashtags like *#jobs* in the profile, in the tweet text, or in both. We also observed individual physicians in certain specialties, such as plastic surgery, cosmetic surgery, and dermatology, using Twitter to market their practice, further reinforcing the fact that Twitter is now accepted as a mainstream platform that physicians can use to communicate directly with patient populations.

Finally, mental health and well-being were consistently ranked in the top 50 topics that US physicians discussed on Twitter. Across a multitude of hashtags, including *#mentalhealth*, *#wellness*, *#depression*, and *#mindfulness*, US physicians used Twitter to engage in dialogue about wellness. These trends may be particularly useful for monitoring physicians’ well-being at a local, regional, or national level, since the mental health of clinicians is notoriously challenging to measure on a regular basis. We hypothesize that during stressful events, such as the COVID-19 pandemic, Twitter may offer unprecedented insight into the overall mental health status of physicians, and we are following up this study with one involving a sentiment analysis of all physician tweets that have been posted during the pandemic.

### Challenges, Strengths, and Limitations

During the planning of this study, it was clear to us how challenging it would be to identify physicians on Twitter, hence the lack of publications on this topic in the literature. Twitter was designed to be fast and easy with regard to sign-up and use, and the consequence of this focus on user experience is that there are very few denominators for identifying subgroups within Twitter. The most challenging task of this study was identifying US physician tweets (n=6,399,146) from the trillions of tweets generated worldwide.

Although numerous studies have analyzed the Twitter activity of the entire Twitter population, the complexity of this task increases significantly once subgroups need to be identified and analyzed. We believe that the methodology outlined in this study offers a robust, scalable, and repeatable method of identifying physician accounts on Twitter. We also recognize that this approach is not perfect and hope that this study serves as a foundation for future work to further improve the identification of physician accounts for future analyses.

The computational requirements for mining approximately 1 trillion tweets and analyzing millions of tweets should not be underestimated. Once the data set reaches beyond a few months of Twitter data, the data set is too large for manual assessment. As such, algorithmic approaches to filtering and analyzing the data are recommended. Analyzing this amount of data often exceeds the computational limits of a typical laptop or desktop, and more high-performance graphics processing units or high-performance computing clusters in the cloud are recommended to analyze the data in a reasonable amount of time, particularly if a real-time analysis is desired.

This study had 3 main limitations. First, all physicians who were identified as active users on Twitter were self-reported physicians. One’s status as a physician can be very difficult to validate unless this information is cross-referenced against state licensing data. Physicians with verified accounts on Twitter (ie, accounts with blue badges) are the most reliable, since Twitter has a rigorous verification process that involves checking credible sources, such as health system websites and media sources, and using other means for verification [28]. Since activity trends were similar between the nonverified and verified accounts, we are reasonably confident that our approach produced a reliable cohort of physicians. Second, while Twitter’s official verification process via the blue badge offers the most reliable method for validating physician accounts within pragmatic means, this approach has its limitations. Twitter has accidentally verified fake accounts, has temporarily halted verifications multiple times to improve their internal processes, and has overall been slow to verify physicians—an issue Twitter has recently addressed during the COVID-19 pandemic [37-40]. Third, it is very likely that our report underestimates the number of active US physicians on Twitter because some physicians may not indicate their status as a clinician. Although we explored various methodologies for matching Twitter accounts, such as using state licensing data, these approaches were not practical for this study, and they will be considered for future investigations.

### Future Directions

There are many opportunities to build on this study. We are planning future studies to focus on specific events, such as the COVID-19 pandemic. For example, by segmenting the physician tweets by state and correlating them with epidemiological data, we plan to study whether there was increased physician activity during pandemic surges. Similarly, machine learning and statistical analyses could be used to explore whether physicians’ Twitter activity can be used as a predictor for public health alerts in the future. Physician activity could also be used as a potential real-time barometer for physician well-being at a regional or national level; by applying natural language processing techniques, such as sentiment analysis, such a data set could provide a baseline measurement of physician morale at any given point in time. We are exploring more in-depth analyses of Twitter bios; topic modeling; and the correlation of self-reported identities with validated physician registries, such as medical licensing boards. Finally, we feel that our methodology and this first comprehensive study of physician activity on Twitter offer a foundation to building more accurate and precise algorithms for identifying physician accounts nationally and globally. We also provide a more complete characterization of the social media activity of US physicians than what has been possible in the past.

### Conclusion

More physicians are using Twitter and covering a wider range of topics in their tweets. Twitter is entering a new phase of mainstream use among physicians, even though physician activity still represents a tiny fraction of total Twitter activity. These trends are evidence that Twitter can be a valuable source of data that can be used to understand social trends, interests, and the perceptions of the physician community. Unlike the more stringent settings of clinical practice, physicians are opening up on social media and showing a different side of themselves that is typically protected in the professional clinical setting. Physicians on Twitter may be offering a unique, more comprehensive view into the physician psyche, making Twitter an intriguing platform for further study and exploration.

## Appendix

Multimedia Appendix 1 Boolean logic for identifying physician accounts on Twitter.

Multimedia Appendix 2 Top 100 tweeted hashtags and themes between 2016 and 2020.

Multimedia Appendix 3 Word clouds for hashtags from 2016 to 2020.
