# Supplementary figures and images for: Twitter Usage Among Physicians From 2016 to 2020: Algorithm Development and Longitudinal Analysis Study
Source: J Med Internet Res. 2022 Sep 6;24(9):e37752. doi: 10.2196/37752 (PMC9490540; doi:10.2196/37752)

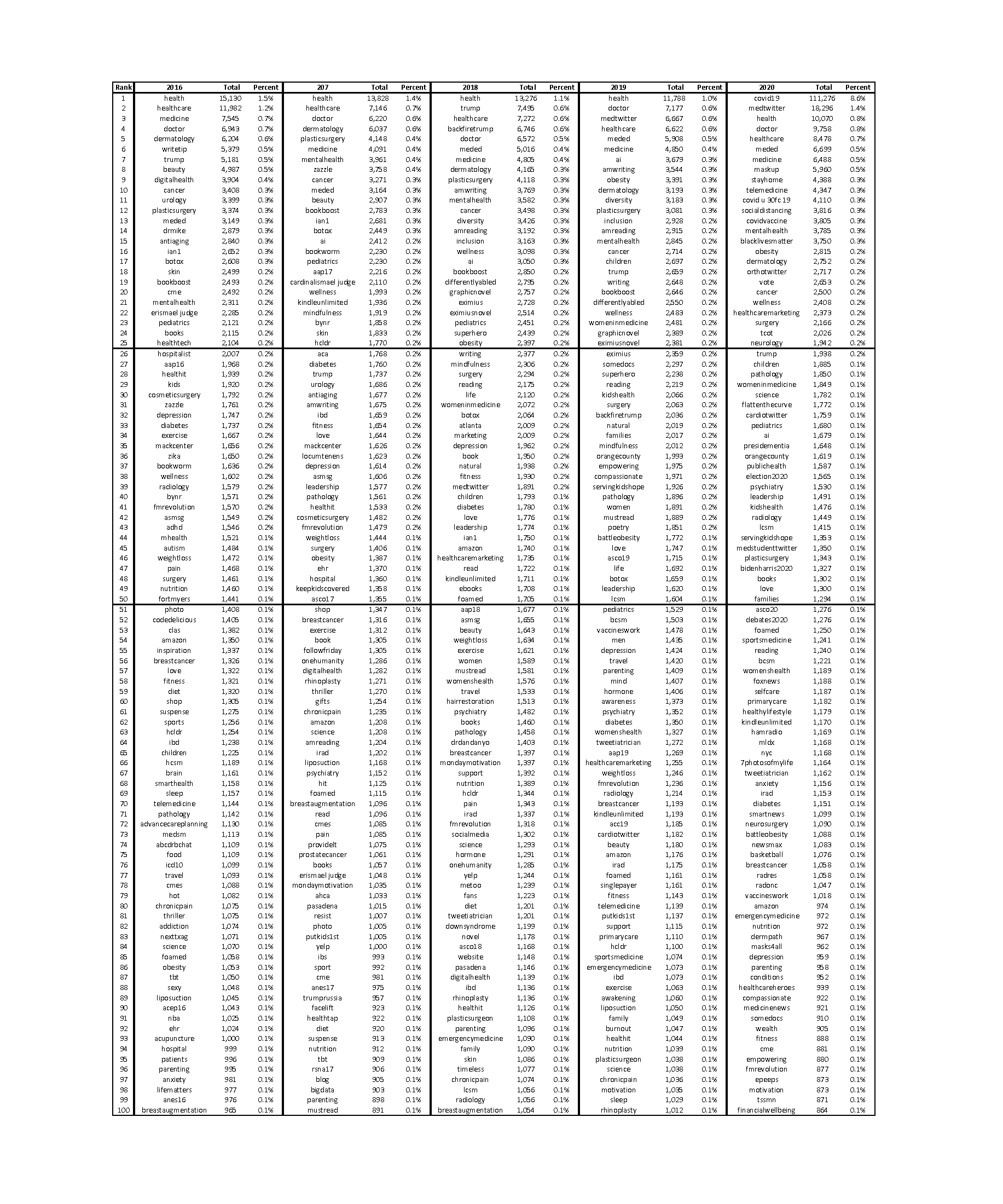

Supplement: Multimedia Appendix 2 [file jmir_v24i9e37752_app2.png]

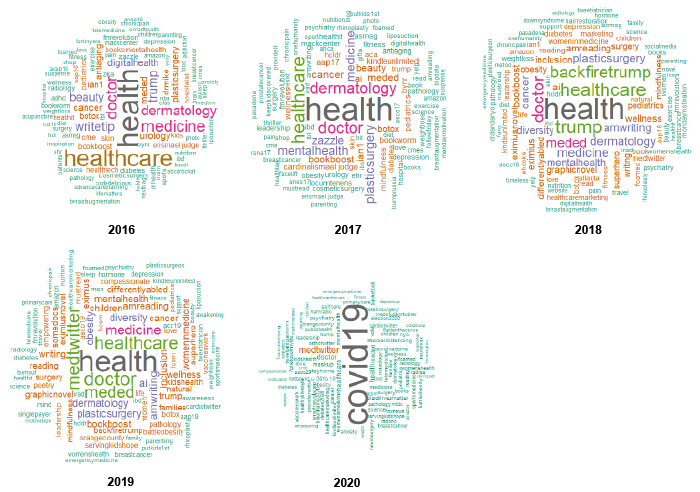

Supplement: Multimedia Appendix 3 [file jmir_v24i9e37752_app3.png]
